# Structural mechanism of the enhanced glass-forming ability in multicomponent alloys with positive heat of mixing

**DOI:** 10.1038/srep38098

**Published:** 2016-11-29

**Authors:** S. Y. Wu, S. H. Wei, G. Q. Guo, J. G. Wang, L. Yang

**Affiliations:** 1 College of Materials Science and Technology, Nanjing University of Aeronautics and Astronautics, Nanjing, 210016, P.R. China; 2 Department of Microelectronic Science and Engineering, Faculty of Science, Ningbo University, Ningbo, 315211, P.R. China; 3 School of Materials Science and Engineering, Anhui University of Technology, Ma’anshan, 243002, China

## Abstract

The issue, microalloying certain element with positive heat of mixing leading to the enhanced glass forming ability (GFA) in multicomponent alloys, has been investigated by systematic experimental measurements coupled with theoretical calculations. It is found that in the Nb-doped CuZr alloys, strong interaction between Nb and Zr atoms leads to a shortened pair distance. In addition, fraction of the icosahedral-like local structures increases with Nb addition and Nb solutes are apt to be separated with each other. These factors contribute to an increase of the atomic level efficiency to fill space and formation of the short-to-medium range orderings. As a result, the amorphous structure is stabilized and the GFA is enhanced accordingly. This work provides an in-depth understanding of microalloying-induced high GFAs in multicomponent alloys and is helpful for guiding the development of more metallic glasses with high GFAs via microalloying, despite the positive heat of mixing between the constituent elements.

In principle, liquid cooled as rapidly as possible can transform into solid having glassy structure. As a result, a mass of glassy alloys have been successfully fabricated by cooling the melts rapidly^1^,^2^,^3^,^4^. However, so far, the cooling rate cannot be high enough to guarantee the glass formation in any alloy composition under laboratory conditions. Therefore, the critical casting size of amorphous alloys is usually regarded as a significant laboratory indicator of the glass forming ability (GFA). According to the “confusion principle”^5^, metallic glasses (MGs) with relatively large critical casting sizes can be prepared in some multicomponent systems. In particular, it has been well accepted that microalloying certain element in some binary mother alloys can greatly enhance their GFAs^6^, providing a feasible strategy for developing more multicomponent MGs with relatively large critical casting sizes.

According to an empirical criterion proposed by Prof. Inoue, MGs usually can be developed in alloy system where there is negative heat of mixing among the constituent elements^7^. However, recently, it is surprising that a range of multicomponent bulk metallic glasses (BMGs) have been successfully fabricated by applying microalloying technique in Zr-based^8^, Cu-based^9^, Fe-based^10^, and Ni-based^11^ alloy systems, where the minor element has a positive heat of mixing (PHM) with the main constituent element. This implies that besides the requirement of negative heating of mixing among the constituent elements to ensure high GFA in many alloy compositions, choosing certain minor element possessing a PHM with the main constituent element is another useful strategy for developing more multicomponent BMGs. For this purpose, understanding the underlying mechanism of the enhanced GFA induced by microalloying the element with PHM is required^12^.

It is an interesting phenomenon that there is a very narrow composition range (usually about 1–2 at.%) enabling formation of BMGs when PHM exists in alloys. In other words, the GFA is very sensitive to the concentration of the minor element. It is much more difficult to reveal the underlying mechanism of glass formation in this type of BMGs, by using the existing criteria or rules^13^,^14^,^15^,^16^,^17^. Fortunately, revealing the mechanism of the glass formation in alloys from the structural perspective is an effective approach and has been pursued for decades^18^,^19^,^20^,^21^,^22^,^23^. In particular, recently, the structural mechanism of microalloying effect on the GFA has been studied in many multicomponent alloys^24^,^25^,^26^,^27^,^28^,^29^. Therefore, this issue is expected to be addressed by studying the atomic-to-cluster level microstructure.

CuZrNb alloys are selected as the research prototypes in the present work, due to the following reasons: 1) Nb element has PHM values with both Cu and Zr which are the main constituent elements^30^,^31^; 2) this ternary system has a narrow composition range (1–2 at.% Nb) forming the glassy alloys with a relatively large critical casting size^9^,^32^. By applying synchrotron radiation-based experiments coupled with simulations (such as Reverse Monte-Carlo) and calculations (such as electronic density of state), we reveal the structural mechanism which can explain how minor Nb addition contributes to the increase of GFA in the ZrCu alloy system.

## Results and Discussion

### The amorphous structure of samples

**Figure 1:**
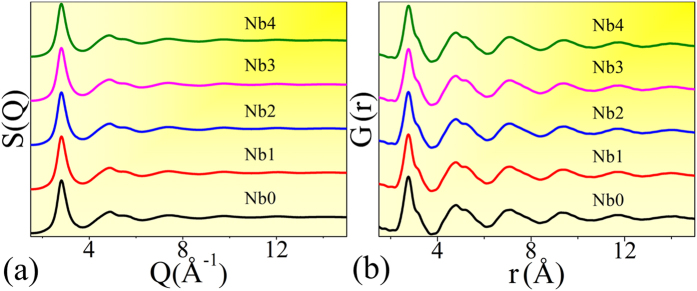
(**a**) The structural factor (S(Q)) and (**b**) the total pair distribution function (G(r)), which are the one-dimensional data transformed from a two-dimensional diffraction pattern obtained from the synchrotron radiation measurement. Nb_x_ (x = 0, 1, 2, 3, and 4) denote the (Cu_64_Zr_36_)_100−x_Nb_x_ (x = 0, 1, 2, 3, and 4) compositions.

Figure 1 shows the structural factor (S(Q)) and the total pair distribution function (G(r)), which are the one-dimensional data transformed from a synchrotron radiation-based two-dimensional diffraction pattern. The absence of any sharp peaks behind the first strong peak in the S(Q) curves and the smooth oscillations in the G(r) data adequately indicate the full amorphous structure in all the selected samples^28^.

**Figure 2:**
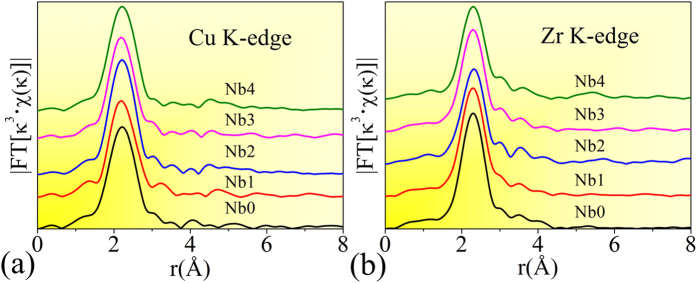
Radial distribution function (RDF) curves for (**a**) Cu- and (**b**) Zr K-shell absorption edges, which are obtained by reverse Fourier transforming the EXAFS signal into real space. Nb_x_ (x = 0, 1, 2, 3, and 4) denote the (Cu_64_Zr_36_)_100−x_Nb_x_ (x = 0, 1, 2, 3, and 4) compositions.

In addition, the radial distribution functions (RDFs) of Cu and Zr K-edge obtained from the synchrotron radiation-based extended X-ray absorption fine structure (EXAFS) experiments are plotted in Figure 2(a) and (b), respectively. It is shown that there is one main peak denoting the first-shell atomic distribution around Zr or Cu centers. This is a specific indicator of the amorphous structure in alloys^33^. There are slight shape and intensity differences in Cu or Zr K-edge RDFs among all the samples, implying some fines structural changes. However, we cannot obtain their atomic-scale differences by directly studying these EXAFS signals, because that co-existing of various clusters is the intrinsic feature in amorphous alloys^21^, making it impossible to directly fit Cu or Zr EXAFS RDF in MGs by only using one structural model.

### Success of the Reverse Monte-Carlo (RMC) simulation

**Figure 3:**
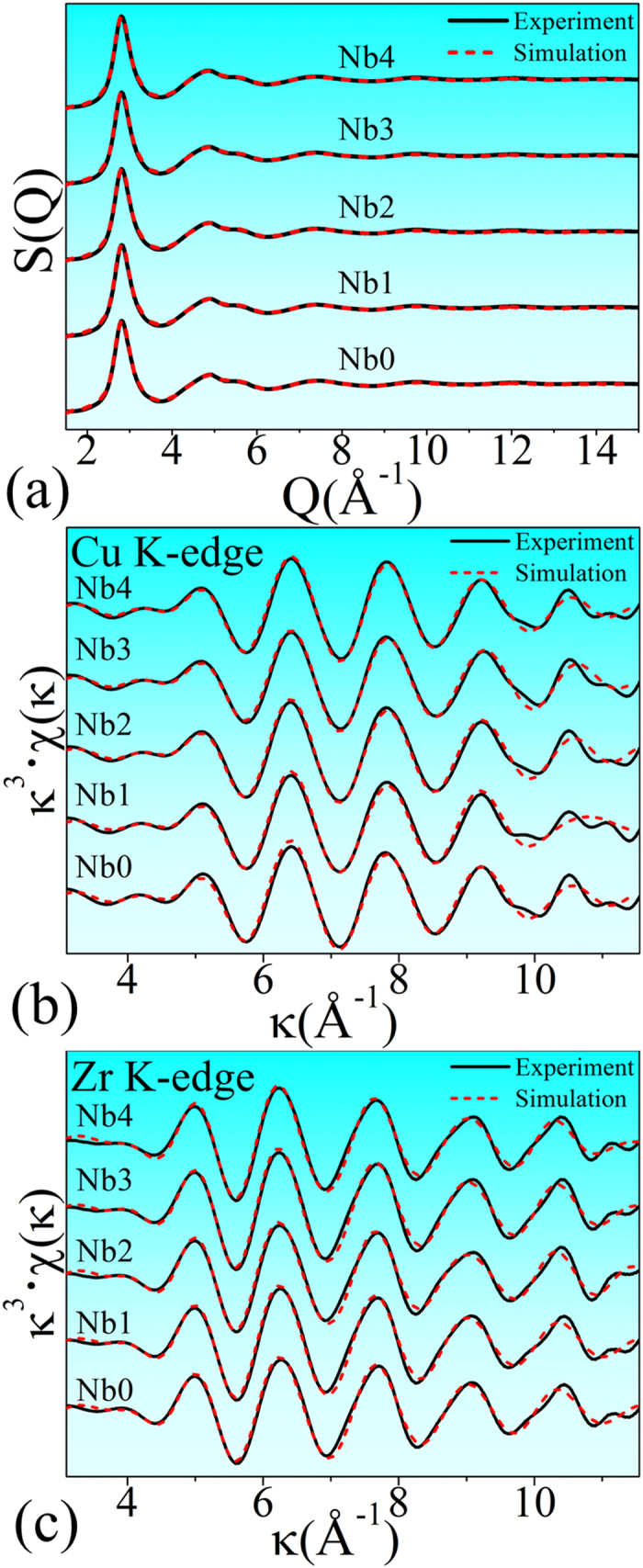
RMC simulation result, including (**a**) XRD S(Q) factor, (**b**) Cu K-edge, and (**c**) Zr K-edge EXAFS spectra. The solid and the dashed lines denote the experimental and the simulation data, respectively. Q is the transfer of wave vector in reciprocal space. κ and χ(κ) represent the photoelectron wave vector and the κ-space EXAFS signal, respectively. Nb_x_ (x = 0, 1, 2, 3, and 4) denote the (Cu_64_Zr_36_)_100−x_Nb_x_ (x = 0, 1, 2, 3, and 4) compositions.

Figure 3 shows the synchrotron radiation-based X-ray diffraction (XRD) and EXAFS experimental data, and their simulated curves. The good matching between the experimental/simulated pairs indicates the success of RMC simulation. It should be noted that although during RMC simulation, the simulated experimental data do not include that of Nb K-edge EXAFS, we still can get reliable structural information because of the following reasons: 1) EXAFS is an element-specific method available for measuring the surroundings of each kind of atoms. In other words, all the neighbour atoms around each atom can be elementally distinguished when EXAFS data is fitted or simulated^34^. Since Cu and Zr EXAFS data can reflect all of their neighbourhood information containing Cu, Zr, and Nb atoms, how the Nb atoms distribute around Cu or Zr centers also can be probed; 2) one set of XRD and two sets of EXAFS (Cu and Zr) were simultaneously simulated in this work. During the RMC simulation, all of these experimental data should fit with their counterparts calculated theoretically from the same structural model. Such constraint can eliminate the computational randomness. Compared with directly fitting the EXAFS signals using one cluster model which usually is applied for crystals, the RMC simulation provide a more reliable structural model which can reflect the intrinsic structural features in glassy alloys.

### Atomic-level structural information

#### Band shortening of Zr-Nb

Some atomic-scale structural information could be deduced from the RMC simulated structural models, including the first-shell coordination numbers (CNs) and the atomic pair distances. They are listed in Tables 1 and 2. It is found that the CN values around the large atom (Zr) and the small atom (Cu) barely change with the compositional variation, i.e., Zr and Cu have the CN values about 14 and 11 in all these CuZr or CuZrNb samples, respectively. It is interesting that the CN values around the Nb centers are about 12 when microalloying 1 to 2 at.% Nb in the CuZr alloys. This implies that compared with Zr or Cu centers, Nb atoms are more likely to be centers in icosahedral clusters.

Concerning the atomic pair distances in (Cu_64_Zr_36_)_99_Nb_1_, it is observed that atomic pairs between the main constituents such as Zr-Zr, Zr-Cu, and Cu-Cu do not have obvious shorter or longer bonds compared with their sum of Goldschmidt atomic radii, and these values barely change with the composition. However, it is worth noting that there is a remarkable bond shortening in the Zr-Nb pair, which is about 0.2 Å shorter than its corresponding Goldschmidt value. It suggests that there is a relatively strong interaction between Zr and Nb atoms. This is rather surprising because Zr-Nb has a PHM^31^ and no Zr-Nb binary crystalline phase could be formed under ambient conditions^35^.

#### Nb-(Cu, Zr, or Nb) connections

**Figure 4:**
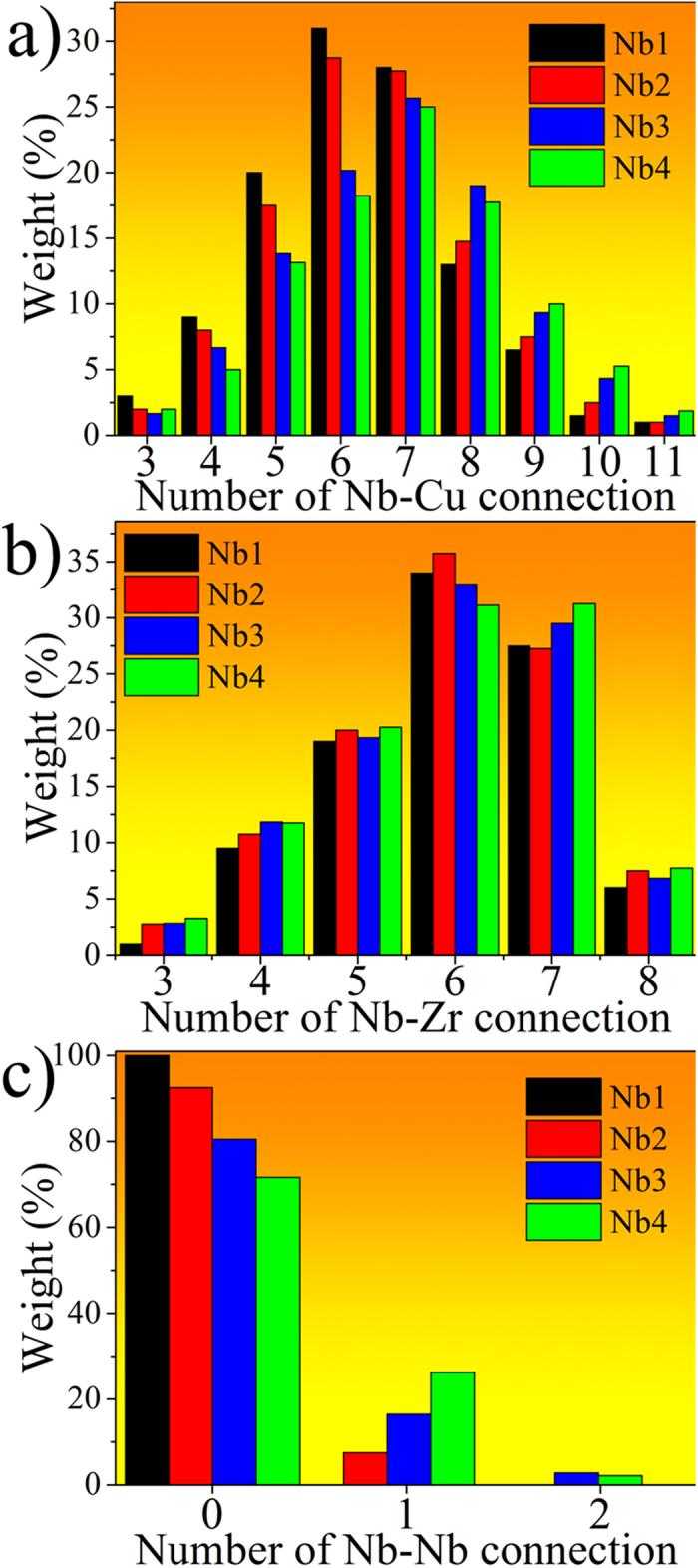
Distributions of Nb-centered connections, including. (**a**) Nb-Cu, (**b**) Nb-Zr, and (**c**) Nb-Nb atomic pairs. Nb_x_ (x = 1, 2, 3, and 4) denote the (Cu_64_Zr_36_)_100−x_Nb_x_ (x = 1, 2, 3, and 4) compositions.

Since minor Nb addition tunes the GFAs in ZrCu alloys, studying the Nb-connected atomic structure is required. All the Nb-atom connections are sorted out from the structural model, and the distributions are plotted in Fig. 4. It is observed that there are no obvious differences of the Zr neighbours around the Nb centers among these CuZrNb samples, and in particular 5–7 Zr connections are the most popular ones. This indicates that Nb has almost the same average number (5.9, refers to Table 1) of Zr neighbours despite the change of composition, probably because that the relatively strong Zr-Nb atomic interaction makes the distribution of Zr around Nb unchangeable. Regarding the Nb-Cu pairs in these ZrCuNb alloys, it is obvious that less Cu neighbours are apt to surround the Nb centers in the (Cu_64_Zr_36_)_99_Nb_1_. In particular, 4–6 and 8–10 Cu connections have relatively high and low fractions in this composition, respectively. This indicates that unlike the Nb-Zr pairs, the number of Nb-Cu connections is much more sensitive to the compositional variation.

Although the Nb concentration is rather low in these CuZrNb samples, the numbers of Nb-Nb pairs have been successfully calculated from the RMC simulated structural model. It is shown that Nb atoms do not connect with each other when only microalloying 1 at.% Nb in the CuZr alloy, while a small amount of 1 and even 2 Nb-Nb connections appear when 3–4 at.% Nb is added. This suggests that separated Nb atoms are more favored in microstructure of MGs with larger GFA, which is consistent with the viewpoint that solutes should avoid direct connection in the efficient packing model^36^. Although in previous work, it is revealed that chemical heterogeneity in MGs leads to strings connecting the minor atoms (the solutes)^37^, such strings are absent in the (Cu_64_Zr_36_)_99_Nb_1_ which has the local GFA maximum. It has been proposed that solute-solute strings could not appear until the alloy composition has excess solutes^21^. In other words, the relatively low concentration of minor element leads to avoidance of the direct connection between solutes. In this sense, it is reasonable that in the (Cu_64_Zr_36_)_99_Nb_1_, Nb atoms (the solutes) are separated with each other and more likely to play a role of glue atoms^38^,^39^ rather than strings, connecting their neighbouring Zr and Cu atoms (the solvents).

### Cluster-level structural information

#### Distribution of clusters

**Figure 5:**
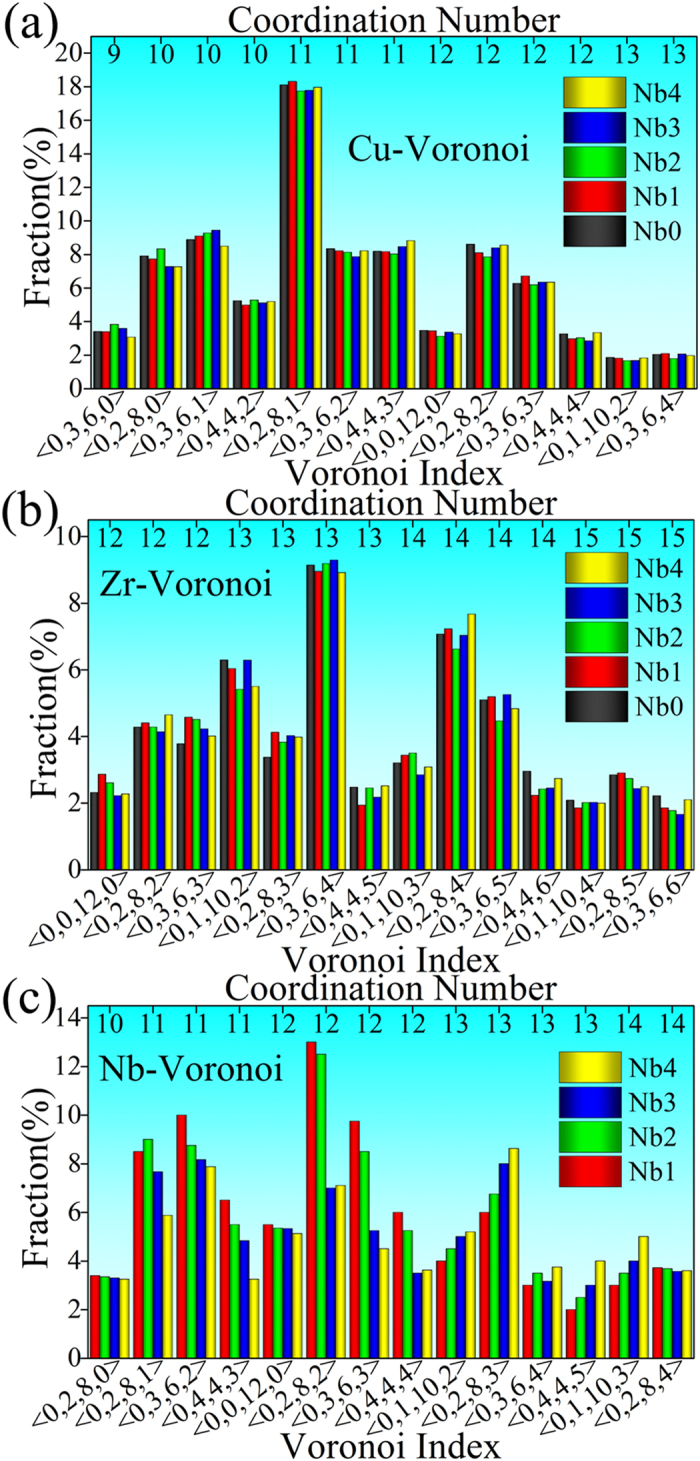
Distribution of the major VCs centered with (**a**) Cu, (b) Zr, and (**c**) Nb atoms. Coordination numbers here are the numbers of shell atoms surrounding the centers in VCs. Note only those whose fractions are larger than 1.5% are selected. Nb_x_ (x = 0, 1, 2, 3, and 4) denote the (Cu_64_Zr_36_)_100−x_Nb_x_ (x = 0, 1, 2, 3, and 4) compositions.

Furthermore, studying cluster-level information is required. Via the Voronoi tessellation, all the Voronoi clusters (VCs) could be extracted and indexed, whose distributions are plotted in Fig. 5. It is known that CuZr is a typical binary alloys system enabling glass formation with a quite wide composition range, and the mother alloy Cu_64_Zr_36_ used in this work is a representative composition with a relatively large GFA^40^. As shown in Fig. 5, the distributions of VCs with Cu or Zr centers do not vary much among these CuZr or CuZrNb samples. The most popular Cu- and Zr-centered VCs are indexed as <0,2,8,1>, <0,2,8,2>, <0,3,6,2>, <0,4,4,3>, <0,3,6,1>, and <0,3,6,4>, <0,1,10,2>, <0,2,8,2>, <0,3,6,3>, <0,2,8,4>, respectively. These major VCs have CNs (the number of the shell atoms) ranged from 11 to 14.

<0,0,12,0> VC is an ideal icosahedron, which also is called as full icosahedron. <0,0,12,0> is expected to be the popular short-range ordering contributing to the glass formation in amorphous alloys^41^,^42^. In particular, it is proposed that high fraction of full icosahedron relates to high GFA in CuZr binary system^43^,^44^. In this work, for the Cu-centered clusters, the <0,0,12,0> has a relatively low concentration while another VC, <0,2,8,1>, has a high weight. This is because that some <0,0,12,0> VCs have abnormal or small triangles faces on their surfaces while a <0,0,12,0> should have high geometrical regularity and similar triangles faces in it surface, so that one atom is usually removed from these clusters to get rid of the abnormal or small triangles faces^21^, and then their indices change from <0,0,12,0> to <0,2,8,1>. In fact, the total fraction of the indexed <0,0,12,0> and those changed into <0,2,8,1> has a relatively high value. In addition, some of the Cu- or the Zr-centered major VCs (such as <0,2,8,2>, <0,3,6,3>, and <0,4,4,4>) can be regarded as icosahedral-like clusters, because they have similar geometrical configurations with <0,0,12,0> and also contain abundant fivefold symmetrical features^45^. For instance, <0,2,8,2> has 20 triangle faces on its surface which is the basic geometrical feature of an icosahedron, so that <0,2,8,2> could be called as an icosahedral-like (or distorted icosahedral) cluster. In addition, <0,2,8,2> has 8 fivefold, 2 fourfold, and 2 sixfold point symmetries, indicating that the fivefold symmetry has a high weight of 73%.

We stress that it is the Nb addition that leads to the enhancement of GFA. Therefore, studying the role of Nb element at the cluster level in particular the Nb-centered clusters also is useful. It is worth noting that compared with the Zr- or Cu-centered counterparts, there is a higher fraction of these icosahedral-like Nb-centered VCs, as shown in Fig. 5c. In previous work, it has been proposed that icosahedral clusters are favored in the microstructure of glassy alloys^46^,^47^. In this sense, it is reasonable that the Nb addition increasing the icosahedral-like clusters contributes to a high GFA in the ternary alloys. In addition, as listed in Table 1, the CN of Nb atoms is about 12 in the (Cu_64_Zr_36_)_99_Nb_1_, while the counterparts of Zr and Cu are 14 and 11, respectively. This is consistent with the cluster information obtained here.

#### Electronic basis of bond shortening

In general, bond shortening is often observed in many intermetallic crystalline compounds and even some glassy alloys^25^,^26^,^29^,^48^. Nevertheless, bond shortening between Zr-Nb pair possessing a PHM is a surprising observation in this work and should be explained.

**Figure 6:**
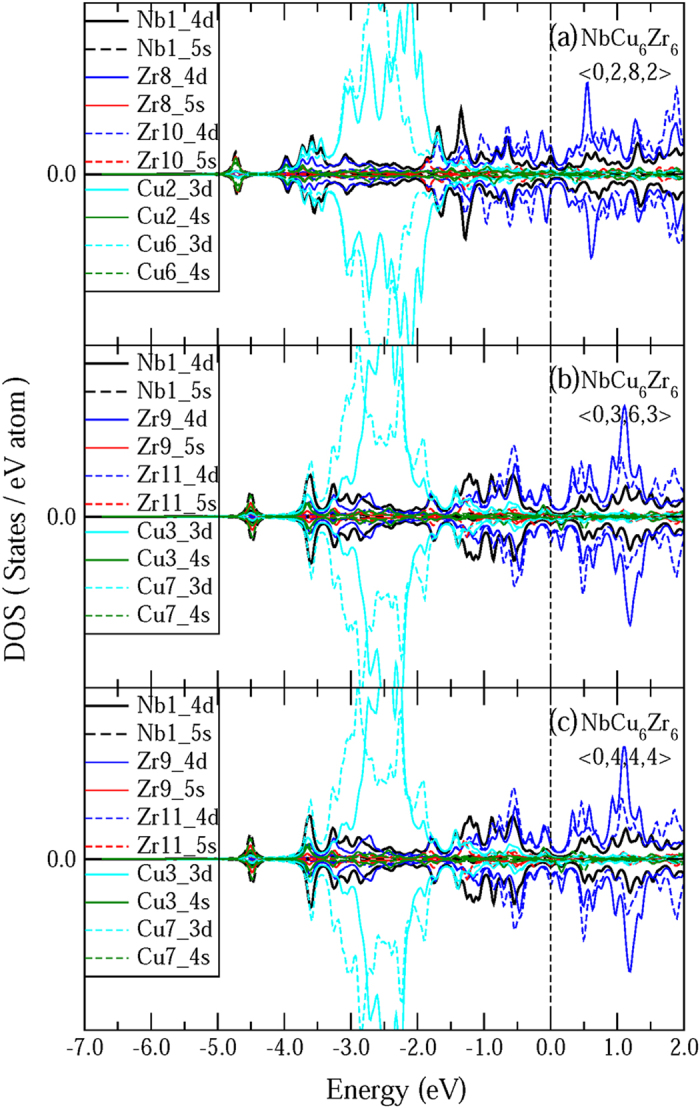
The partial electronic density of state (DOS) for three typical Nb-centered icosahedral-like VCs, which are indexed as <0,2,8,2>, <0,3,6,3>, and <0,4,4,4>. Only some representative atoms are plotted. The number behind each atom denotes the serial number in these clusters. The Fermi energy level (*E*_F_) possesses a position of 0 eV.

Revealing the electronic basis is helpful for understanding the origin of bond shortening in the Zr-Nb atomic pair. The DMol3 cluster method was performed based on the density functional theory^49^ under the generalized gradient approximation^50^. Unlike the crystalline alloys which have the determined unit cells which can be stacked periodically to form the long-rang ordering, the corresponding glassy alloys only possess the short-range ordering. It is difficult to calculate the electronic density of state (DOS) for all the clusters in the microstructure of glassy alloys, because there are dozens kinds of VCs. In this work, it is revealed that some Nb-centered icosahedral-like VCs with a relatively high total fraction contribute to the enhancement of GFA. Therefore, three typical Nb-centered icosahedral-like VCs, indexed as <0,2,8,2>, <0,3,6,3>, and <0,4,4,4>, were selected to perform the calculation of DOS. The partial DOS calculated from some representative atoms are shown in Fig. 6. From Fig. 6(a), it is found that the partial DOS of Nb-4d and Zr-4d are mainly distributed around the Fermi level (from −4.0 eV to 2.0 eV). On the other hand, the partial DOS of Cu-3d are mainly ranged from −3.8 eV to −1.5 eV, where there only are some minor partial DOS distributions for Nb-4d and Zr-4d. For the <0,2,8,2> cluster, there is weak orbital hybridization between Nb-5s and Cu-4s or Nb-4d and Cu-4s at the energy range from −5.0 eV to −4.0 eV, while there is noticeable orbital hybridization between Nb-4d and Zr-4d, as shown in Fig. 6(a). The same results are observed in other Nb-centered VCs indexed as <0,3,6,3> and <0,4,4,4>, as shown in Fig. 6(b) and (c). Therefore, strong hybridization interactions between the Nb centers and their neighbours in particular the Zr atoms are confirmed in these Nb-centered icosahedral-like VCs. This can be extrapolated to explain the bond shortening of Nb-Zr atomic pairs in the ZrCuNb glassy alloys. In previous work studying CuZrAl glassy alloy^25^, it was found that strong hybridization interaction between CuAl pair is the origin of the shortened CuAl bond.

#### Packing inside clusters

**Figure 7:**
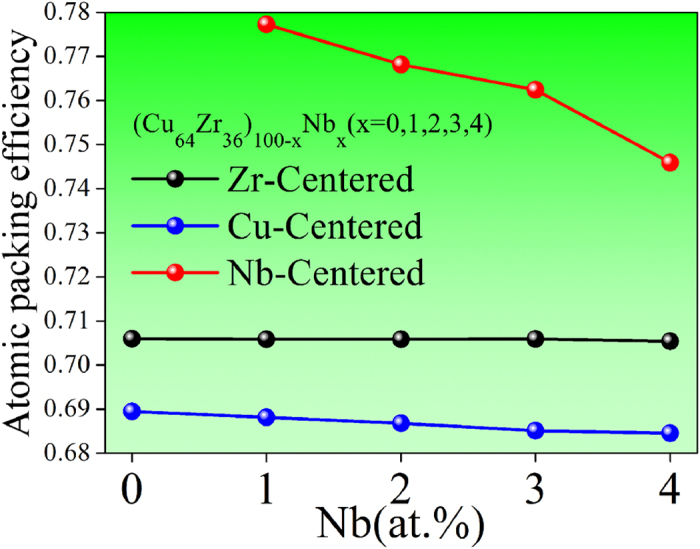
The atomic packing efficiencies (APEs) deduced from Cu-, Zr-, and Nb-centered VCs.

In our previous work, it has been pointed out that the atomic packing efficiency (APE) inside clusters is a structural parameter which strongly relates to the GFA in binary alloys^51^. It accords with the dense packing principle accepted widely^52^. Therefore, in this work studying the multicomponent MGs, the APE also is calculated, by the following equation


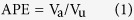


where V_a_ and V_u_ denote the volume of the embedded atoms inside clusters and the total volume of clusters, respectively. The APE values of Cu-, Zr-, and Nb-centered VCs for all the samples are plotted in Fig. 7. It is shown that the APE values of Cu- and Zr-centered VCs do not obviously change with the composition. This is because that 1–4 at.% minor Nb addition could not significantly affect the local structures centered with Cu or Zr solvents. This has been confirmed by the cluster-scale structural information shown in Fig. 5.

Concerning the Nb-centered VCs, we notice that the APE value increases from 0.74 to about 0.78 when the Nb concentration drops from 4 to 1 percentage. This illustrates that as expected, there is a relatively high APE value in the (Cu_64_Zr_36_)_99_Nb_1_, where there is a local maximum of GFA. In addition, it is interesting that the (Cu_64_Zr_36_)_99_Nb_1_ has a rather high Nb-centered APE of 0.78, much larger than the Cu- and the Zr-centered APE values (0.69 and 0.71), and even larger than that (0.74) of the face centered cubic structure. This is probably because that the strong Nb-Zr interaction induces obvious volume shrinkage of clusters in the (Cu_64_Zr_36_)_99_Nb_1_ (see Supplementary Information file).

### Structural origin of the enhanced GFA despite the PHM

We have obtained abundant atomic-to-cluster level structural information, based on which we can discuss why microalloying Nb in the CuZr alloy enhances the GFA. It has been proposed that in quasicrystals, stacking icosahedra in three-dimensional space will leave some voids^53^. For disorder systems, it is revealed that because the icosahedron is highly close-packed but is difficult to grow, only stacking icosahedra cannot fill space with an efficiency as high as those in corresponding crystalline alloys or quasicrystals, leading to a structural frustration^54^. That is why the medium range structure in MGs usually has more fractal features than that of quasicrystals^55^. However, if both of some icosahedra-like clusters and the ideal icosahedra are the popular structural units, such structural frustration probably is weakened.

It is found that Cu- or Zr-centered icosahedra (<0,0,12,0>) and icosahedral-like clusters with fruitful fivefold symmetrical features can form the structural frame work in both CuZr binary and CuZrNb ternary alloys. These Cu- or Zr-centered icosahedral or icosahedral-like VCs (such as <0,0,12,0>, <0,3,6,3>, <0,2,8,2>, and <0,4,4,4>) may be an important structural factor affecting the GFA, and the largest critical casting size is about 2 mm in diameter for Cu_64_Zr_36_ and Cu_50_Zr_50_ in the ZrCu binary alloys. When microalloying minor (1–2 at.%) Nb in the CuZr mother alloy, more icosahedral-like VCs are formed around the Nb centers, thus, the amorphous microstructure becomes more stable. In addition, it is revealed that the ZrNb bond is shortened due to their strong atomic interaction, leading to a denser atomic packing to fill space^51^. Moreover, Nb atoms are solutes avoiding direct connection between each other, which is helpful for forming short-to-medium range orderings^21^. All of these Nb addition-induced structural changes contribute to the more stable structure and the better GFA.

On the other hand, the GFA is very sensitive to the concentration of Nb^9^. In particular, when more than 3 at.% Nb is added, the GFA is deteriorated. This is because of the structural change induced by the excessive Nb addition. Firstly, compared with the (Cu_64_Zr_36_)_99_Nb_1_, the Nb-Zr pair in the (Cu_64_Zr_36_)_100−x_Nb_x_ (x = 3 or 4) has a longer bond, so that the APE value in the Nb-centered clusters is obviously decreased, leading to a looser packing and less stable structure. Secondly, as shown in Table 1, the solute-solute (Nb-Nb) direct connections appear when microalloying more than 3 at.% Nb in the ZrCu system. Since in a good glass former, the solutes should avoid direct contact^21^,^36^, i.e., no solute-solute connection. Therefore, the solute-solute direct bonding may destroy the short-to-medium range order (the “solutes connecting solvents” mode) which is required for a stable glassy structure. Thirdly, excessive Nb addition tunes the geometrical and chemical features of Nb-centered local structures, leading to a decrease of the icosahedral-like clusters, as shown in Fig. 5c. Due to these structural factors, the amorphous structure is less stable and the GFA is deteriorated accordingly.

## Conclusion

In summary, why BMGs can be formed in spit of the positive heat of mixing is studied from the structural aspect. In particular, the issue that the concentration of Nb affecting the GFA in CuZr alloys is addressed by detecting the atomic- and cluster-level structural factors, via the synchrotron radiation experiments coupled with the RMC simulation. We obtain the following results:

1. Compared with ZrCu binary alloys, more icosahedral-like clusters are formed in the Nb-doped ZrCu alloys, probably due to the modification of atomic size differences (the atomic sizes of Zr, Cu, and Nb atoms are 1.58, 1.28, and 1.46 Å, respectively). This cluster-level structural change can enhance the space filling by stacking both icosahedral and icosahedral-like clusters, contributing to the stabilization of the amorphous structure.
2. When minor Nb atoms (1–2 at.%) are added, Nb-Zr pair has a strong atomic interaction, leading to the shortened Nb-Zr bond and relatively dense atomic packing. In addition, Nb atoms are solutes and apt to be separated with each other. As a result, short-to-medium range order is formed, and the amorphous structure is stabilized accordingly. This is the structural origin that microalloying Nb in the CuZr alloy leads to the enhanced GFA, despite PHM between the Nb and the main constituent elements (Cu and Zr).
3. When excess Nb solutes (>3 at.% in this case) are added, the Nb-Zr bond gets longer so that the atomic packing efficiency for Nb centers decreases. In addition, the appearance of Nb-Nb direct connection retards the formation of short-to-medium range order. This should be the structural reason why excess Nb addition leads to the deterioration of GFA.

## Methods

### Sample preparation

(Cu_64_Zr_36_)_100−x_Nb_x_ (x = 0, 1, 2, 3, and 4) alloy ingots were fabricated by arc melting the constituent elements, Cu [99.9 wt.%], Zr [99.9 wt.%], and Nb [99.9 wt.%] in a Ti-gettered high-purity argon atmosphere. All the ingots were melted at least 5 times to ensure their compositional homogeneity. The corresponding amorphous ribbons were prepared by melt-spinning, producing a cross section of 0.08 × 3 mm^2^.

### Synchrotron radiation-based experiments

Compared with the routine lab experiments (such as the X-ray powder diffraction measurement), the synchrotron radiation-based XRD measurements can provide high-resolution data, which are more reliable for detecting the fine structure features in materials, in particular in amorphous alloys. In this work, the synchrotron radiation-based high-energy (about 100 keV) XRD measurements were performed for all ribbon samples at the beam line, BW5, of Hasylab in Germany. The two-dimensional diffraction patterns were recorded using a Mar345 image plate. Subsequently, because both Cu and Zr have relatively high concentrations in these ribbons, a transmission mode was adopted to measure Cu and Zr K-edge extended EXAFS spectra at the beam lines, BL14W1, in the Shanghai Synchrotron Radiation Facility (SSRF) of China, and U7C, in the National Synchrotron Radiation Laboratory (NSRL) of China. The calculated thicknesses of samples required for Cu and Zr K-edge EXAFS measurements are about 20 and 45 microns, respectively. Therefore, the ribbon samples were polished until their thicknesses were appropriate to measure the Cu K-edge and the Zr K-edge signals. Because Nb K-edge absorption signal overlaps with the background of Zr extended K-edge data, we could not measure the Nb K-edge EXAFS spectra for these samples. Both of the diffraction and the EXAFS raw data were normalized via a standard data-reduced procedure^48^, employing the software PDFgetX^56^ and Visual Processing in EXAFS Researches^57^.

### Reverse Monte-Carlo simulation

RMC-simulation technique is an iterative method for building a structural model in disordered systems with calculated structural information which should agree quantitatively with experimental data (such as the synchrotron radiation-based XRD, EXAFS, and neutron-diffraction data)^58^. In this work, synchrotron radiation-based XRD and EXAFS data were simulated via the RMC method, using the software RMCA^59^. The initial structural models are cubic boxes containing 40,000 randomly distributed Cu, Zr, and Nb atoms, according to the (Cu_64_Zr_36_)_100−x_Nb_x_ (x = 0, 1, 2, 3, and 4) compositions. During each RMC simulation, atoms move randomly within a determined time interval. The experimental data are compared to the simulation ones, based on the iterative calculation^60^:


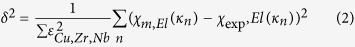


where *δ*^*2*^ represents the deviation between experimental and simulation data, *ε* parameters regulate the weight of the data set given in the fitting procedure, *Ei* indicates Cu, Zr, and Nb elements, and *χ*(κ) parameter is the EXAFS signal. The subscripts “m” and “exp” stand for the simulations and the experiments, respectively. The structural parameter, S(Q), can be obtained by:


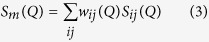


where S_ij_(Q) and w_ij_ are the partial S(Q) and their weights, respectively. S_ij_(Q) can be calculated by:


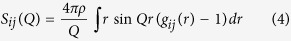


where ρ and r are the average number density and the interatomic distance, respectively. g_ij_(r) is the partial pair distribution function. Thus, the correlation between real space g(r) and reciprocal space S(Q) is presented. The theoretical EXAFS signal χ(*k*), of the ith element is calculated from the following equation:


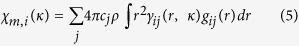


where c_j_ is the concentration of the jth element and γ_ij_ can be calculated by:


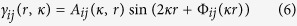


where Aij and Фij denote the amplitude and the phase shift, respectively. They can be obtained by using the FEFF 8.1 code^60^.

After each run, if 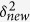
<*δ*^2^, the move is accepted. Otherwise, it is accepted with a probability of exp
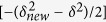
. In this work, to ensure all the atoms can move and avoid strong overlap with neighbour atoms, the cut-off distances between Cu-Cu, Cu-Zr, Cu-Nb, Zr-Zr, Zr-Nb, and Nb-Nb atomic pairs were set to be 2.20 Å, 2.50 Å, 2.25 Å, 2.85 Å, 2.55 Å, and 2.35 Å, respectively. Once all the simulation-experimental pairs fit well, the simulation is accepted and stopped, and the structural model resembling to the microstructure of samples is obtained. Because all the atoms are “frozen” in the RMC simulated model with the determined three-dimensional positions, atomic-to-cluster level structural information could be deduced.

### Voronoi tessellation

Voronoi tessellation is an effective geometrical method for analyzing cluster-level information in disordered materials. According to the Voronoi original algorithm^45^, each convex Voronoi polyhedron (VP) can be built by connecting the perpendicular bisectors between a center atom and all of its neighbouring atoms. Each VP is indexed as <n3, n4, n5, n6,…>, where *ni* denotes the number of i-edged faces on the surface of this polyhedron. Each VP should be embedded in a corresponding convex VC which is made up of one center atom and some neighbouring shell atoms. Thus, ∑ *ni* also stands for the number of the shell atoms in one VC, i.e., the CN around the center atom. The Voronoi algorithm also requires that all VCs should be closed structural units, which can be accomplished by stacking a set of Delaunay tetrahedrons with a shared vertex at the center atom of a VC^61^. This is done so the surface of a VC is only made up of triangular faces, i.e., the VC could be regarded as a deltahedron^21^.

## Additional Information

**How to cite this article**: Wu, S. Y. *et al*. Structural mechanism of the enhanced glass-forming ability in multicomponent alloys with positive heat of mixing. *Sci. Rep.* **6**, 38098; doi: 10.1038/srep38098 (2016).

**Publisher's note:** Springer Nature remains neutral with regard to jurisdictional claims in published maps and institutional affiliations.

## Supplementary Material

Supplementary Information

## Figures and Tables

**Table 1:** Coordination numbers (CNs) around Cu, Zr, and Nb center atoms.

Atomic pairs	CNs (±0.1)				
	Nb_0_	Nb_1_	Nb_2_	Nb_3_	Nb_4_
Zr-Zr	5.2	5.2	5.0	5.0	4.9
Zr-Cu	8.8	8.7	8.6	8.5	8.4
Zr-Nb	—	0.1	0.3	0.4	0.6
Cu-Zr	5.0	4.9	4.9	4.8	4.7
Cu-Cu	6.1	6.1	5.9	6.0	5.9
Cu-Nb	—	0.1	0.2	0.3	0.4
Nb-Zr	—	5.9	5.8	5.7	5.7
Nb-Cu	—	6.1	6.5	6.8	7.0
Nb-Nb	—	0.0	0.1	0.3	0.3
Total of Zr	14.0	14.0	13.9	13.9	13.9
Total of Cu	11.1	11.1	11.0	11.1	11.0
Total of Nb	—	12.0	12.4	12.8	13.0

**Table 2:** First-shell atomic pair distances (APDs) for all the atomic pairs in the selected MGs.

Atomic pair	APD (Å) (±0.01)					D_SGAR_ (Å)
	Nb_0_	Nb_1_	Nb_2_	Nb_3_	Nb_4_	
Zr-Zr	3.20	3.20	3.21	3.20	3.20	3.16
Zr-Cu	2.94	2.94	2.94	2.94	2.95	2.86
Zr-Nb	—	2.85	2.86	2.91	2.92	3.04
Cu-Cu	2.62	2.62	2.62	2.63	2.63	2.56
Cu-Nb	—	2.75	2.76	2.77	2.79	2.74

As a contrast, D_SGAR_ is adopted, which is the interatomic distance calculated from the sum of Goldschmidt atomic radii (SGAR). APDs of Nb-Nb pairs are not listed here due to the uncertainty of these values. Nb_x_ (x = 0, 1, 2, 3, and 4) denote the (Cu_64_Zr_36_)_100−x_Nb_x_ (x = 0, 1, 2, 3, and 4) compositions. The Goldschmidt radii of Zr, Cu, and Nb atoms are 1.58 Å, 1.28 Å, and 1.46 Å^39^, respectively.
