# Supplementary material for: Structural mechanism of the enhanced glass-forming ability in multicomponent alloys with positive heat of mixing
Source: Sci Rep. 2016 Nov 29;6:38098. doi: 10.1038/srep38098 (PMC5126686; doi:10.1038/srep38098)
Supplement: Supplementary Information [file srep38098-s1.docx]

**Structural mechanism of the enhanced glass-forming ability in multicomponent alloys with positive heat of mixing**

S. Y. Wu ^1^, S. H. Wei ^2^**, G. Q. Guo ^1^, J. G. Wang ^3^, and L. Yang ^1 a)^

^1^ College of Materials Science and Technology, Nanjing University of Aeronautics and Astronautics, Nanjing 210016, P.R. China

^2^ Department of Microelectronic Science and Engineering, Faculty of Science, Ningbo University, Ningbo 315211, P.R. China.

^3^ School of Materials Science and Engineering, Anhui University of Technology, Ma'anshan 243002, China

Author to whom correspondence should be addressed: electronic mail: yangliang@nuaa.edu.cn

**Supplementary Information**

**Volume of clusters**

To illustrate that strong Nb-Zr interaction induces volume shrinkage of clusters in the (Cu_64_Zr_36_)_99_Nb_1_, volumes of the Nb-centered Voronoi clusters (VCs) are calculated, whose distributions are plotted in Figure S1. It is shown that the peak position of cluster volume is sensitive to the Nb concentration, that is, when decreasing the Nb concentration from 4, 3, 2, to 1 at.%, the corresponding average volumes drop from 65.7, 65.2, 59.0, to only 58.3 Å^3^.

**

**

Figure S1 Distribution of the volumes of Nb-centered VCs. Nb_x_ (x = 1, 2, 3, and 4) denote the (Cu_64_Zr_36_)_100-x_Nb_x_ (x = 1, 2, 3, and 4) compositions.

**Bond angles inside clusters**

Besides those structural parameters which can be applied for detecting geometrical and topological information, the [bond angle](http://www.baidu.com/link?url=F8UnePVrVOuCeuHszBbKGiztluX3Y7omNNr7Tr2X_VsRmma4nPT-0SfRO9AQIYlQaDPM5fCe7gKyMc6sx8f04fpuLUUck6yq8mvJdHT55NG) inside clusters also is an important structural parameter. The distributions of bond angles inside the Nb-centered VCs are shown in Figure S2. Distributions of bond angels inside Cu- or Zr-centered VCs are not shown here, because it is confirmed that the Nb addition dose not obviously affect them.



Figure S2 Distributions of the bond angles of Nb-centered VCs. Nb_x_ (x = 1, 2, 3, and 4) denote the (Cu_64_Zr_36_)_100-x_Nb_x_ (x = 1, 2, 3, and 4) compositions.

It is found that there is a broad main peak ranged from 40 to 95 degrees for each sample, and the peak position shifts to high degree range when decreasing the concentration of Nb. In general, the smaller the coordination number (CN) of a cluster is, the larger the average bond angle inside this cluster is. This indicates that the (Cu_64_Zr_36_)_99_Nb_1_ should have the smallest average CN value for Nb centers, which [accords with](http://www.baidu.com/link?url=8-xcXCIZsqXNJBMZsa1SW1Fvpn5kFmtVL8pDW5KazUwa9_AU8-M9m6LnpsLhRP_7QKXbkTtatWzFlXSSB-7HSLvE-7YSYLDbDlUP6vxtRza) the data listed in Table 1. In addition, a peak value about 56° is observed in (Cu_64_Zr_36_)_99_Nb_1_. This peak value is very similar with that observed in icosahedral cluters^1^. This is consistent with the analysis of Nb-centered VC in the main body.

**References**

1. Sheng, H.W. et al. Atomic packing and short-to-medium-range order in metallic glasses. *Nature* **439**, 419-425 (2006).
